# Extreme Lateral Interbody Fusion Complicated by Fungal Osteomyelitis: Case Report and Quick Review of the Literature

**DOI:** 10.7759/cureus.2719

**Published:** 2018-05-31

**Authors:** Ronen Blecher, Emre Yilmaz, Marc Moisi, Rod J Oskouian, Jens Chapman

**Affiliations:** 1 Orthopedic Department, Assaf Harofeh, Affiliated to the Sackler School of Medicine, Tel Aviv University, Israel; 2 Swedish Medical Center, Swedish Neuroscience Institute, Seattle, USA; 3 Neurosurgery, Wayne State University School of Medicine., Detroit, USA; 4 Neurosurgery, Swedish Neuroscience Institute, Seattle, USA; 5 Orthopedics Spine Surgery, Swedish Neuroscience Institute, Seattle, USA

**Keywords:** spinal infection, osteomyelitis, xlif, fungal infection

## Abstract

The authors describe a 67-year-old man with a prior history of alcohol abuse who presented with a complaint of worsening low back pain. Four months prior to his presentation, the patient had undergone extreme lateral interbody fusion (XLIF) of his lumbar 3-4 segment for the treatment of his chronic low back and legs pain. Imaging revealed a loosening of his interbody fusion implant on top of his prior lumbar spine instrumentation. In surgery, the removal of his loose implant was followed by decompression, the stabilization of the collapsed segment, and the implant of antibiotic-impregnated polymethyl-methacrylate (PMMA) spacer and beads. At a later stage, the patient underwent an interbody fusion of the affected segment as well as a segmental fusion from T10 to his pelvis. Whereas all aerobes and anaerobes stains were negative for organisms, multiple fungal smears from the failed segment were positive for yeast, and the patient was placed on oral fluconazole. Infections complicating the surgical site of interbody fusions performed by minimally invasive techniques are rare. To the best of our knowledge and after reviewing the literature, this is the first report of an extreme lateral interbody fusion implant complicated by fungal osteomyelitis.

## Introduction

Minimally invasive surgery (MIS) has substantially evolved in recent years, allowing both decompression and stabilization in a variety of conditions affecting the spine [1]. Among the reported advantages of MIS over the traditional open approach is the lower incidence of surgical site infections (SSI), with some reports citing an almost six-fold decrease in the likelihood of acquiring SSI with the former [2]. Regardless of approach, the most commonly cultured pathogen remainsStaphylococcus aureus, affecting more than 50% of all postoperative spine infections [3]. Here, we present a case in which interbody fusion performed using a lateral MIS approach was complicated by fungal osteodiscitis, leading to a septic loosening of the implant.

## Case presentation

History and physical examination

**Figure 1:**
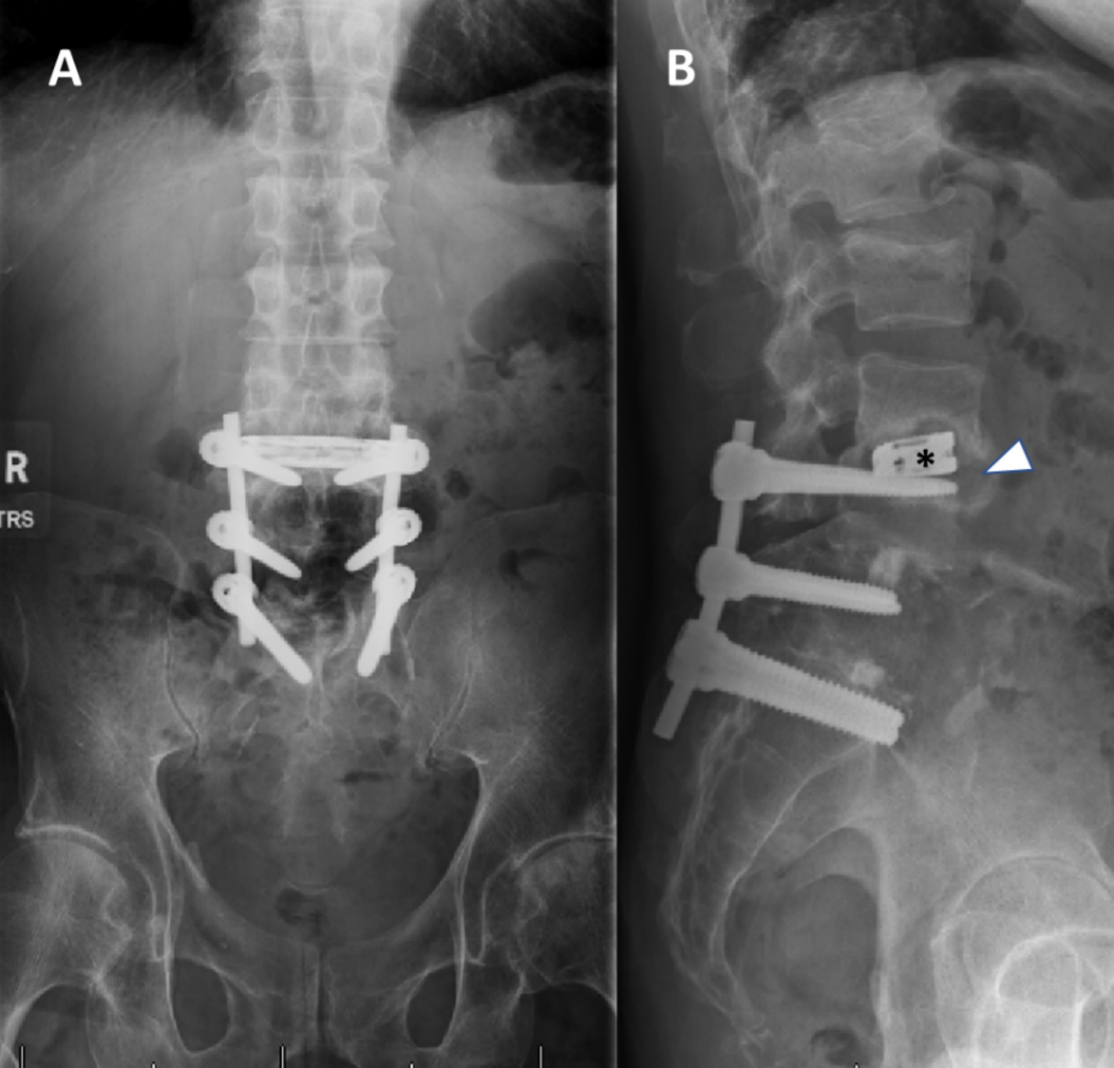
Pre-op AP (A) and lateral (B) lumbar X-rays A previously placed XLIF cage (black asterisk) in the L3-4 disc space is surrounded by a well-demarcated cavitation (white arrowhead). XLIF: extreme lateral interbody fusion; AP: antero-posterior

A 67-year-old male presented to the emergency department with complaints of worsening low back pain and a progressive inability to ambulate as well as to maintain an upright posture. No complaints of fever or bowel and bladder dysfunction were noted. The patient’s past medical history was positive for alcohol abuse and pancreatitis, as well as chronic low back and bilateral leg pain. Relevant past surgical history was positive for prior L4-S1 posterior and interbody fusion performed in 2012 and a recent extreme lateral interbody fusion (XLIF) of L3-4, performed four months prior to his presentation for adjacent segment degeneration and stenosis. The physical exam revealed diffuse weakness, rated 3-4/5 of all bilateral lower extremity key muscles. The workup to rule out infection, including white blood cell count, C-reactive protein (CRP), and erythrocyte sedimentation rate (ESR), was negative. Initial diagnostic imaging consisting of a lumbar x-ray showed that the L3-4 implanted cage has developed significant cavitation around it. In addition, new compression fractures were noted at the vertebral bodies of L1 and L2 (Figure 1).

**Figure 2:**
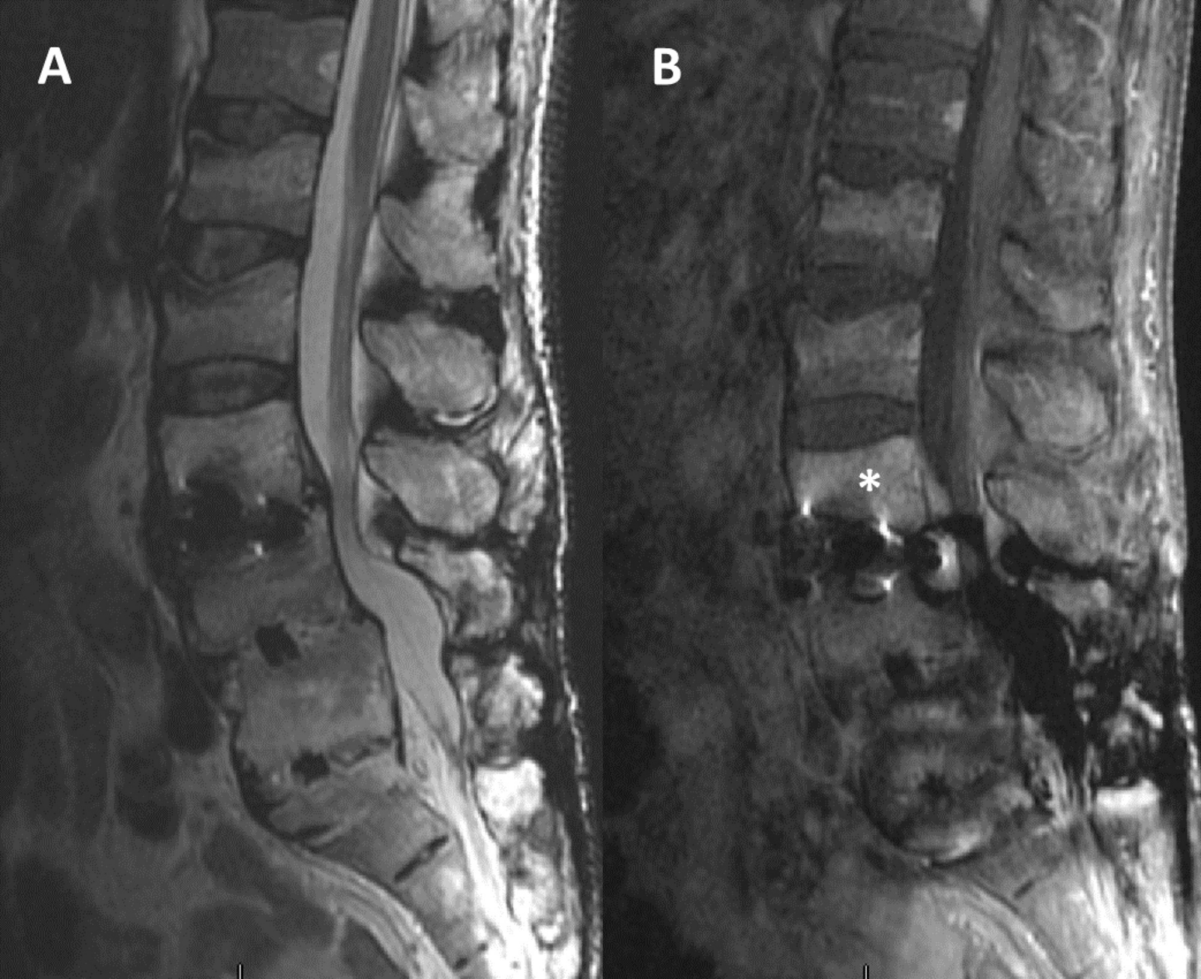
Pre-op lumbar MRI (A,T2 sequence; B, contrast) In addition to adjacent spinal stenosis noted in the T2 sequence (left), the L3 vertebral body shows increased contrast uptake (white asterisk), highly suggestive of infection. MRI: magnetic resonance imaging

Lumbar magnetic resonance imaging (MRI) with contrast demonstrated diffuse edema and enhancement of the L3 and L4 vertebral bodies, strengthening possible infection as the primary etiologic mechanism (Figure 2). Finally, abdominal and pelvic computed tomography (CT) for ruling out a possible intra-abdominal involvement was negative.

Surgical treatment and postoperative course

**Figure 3:**
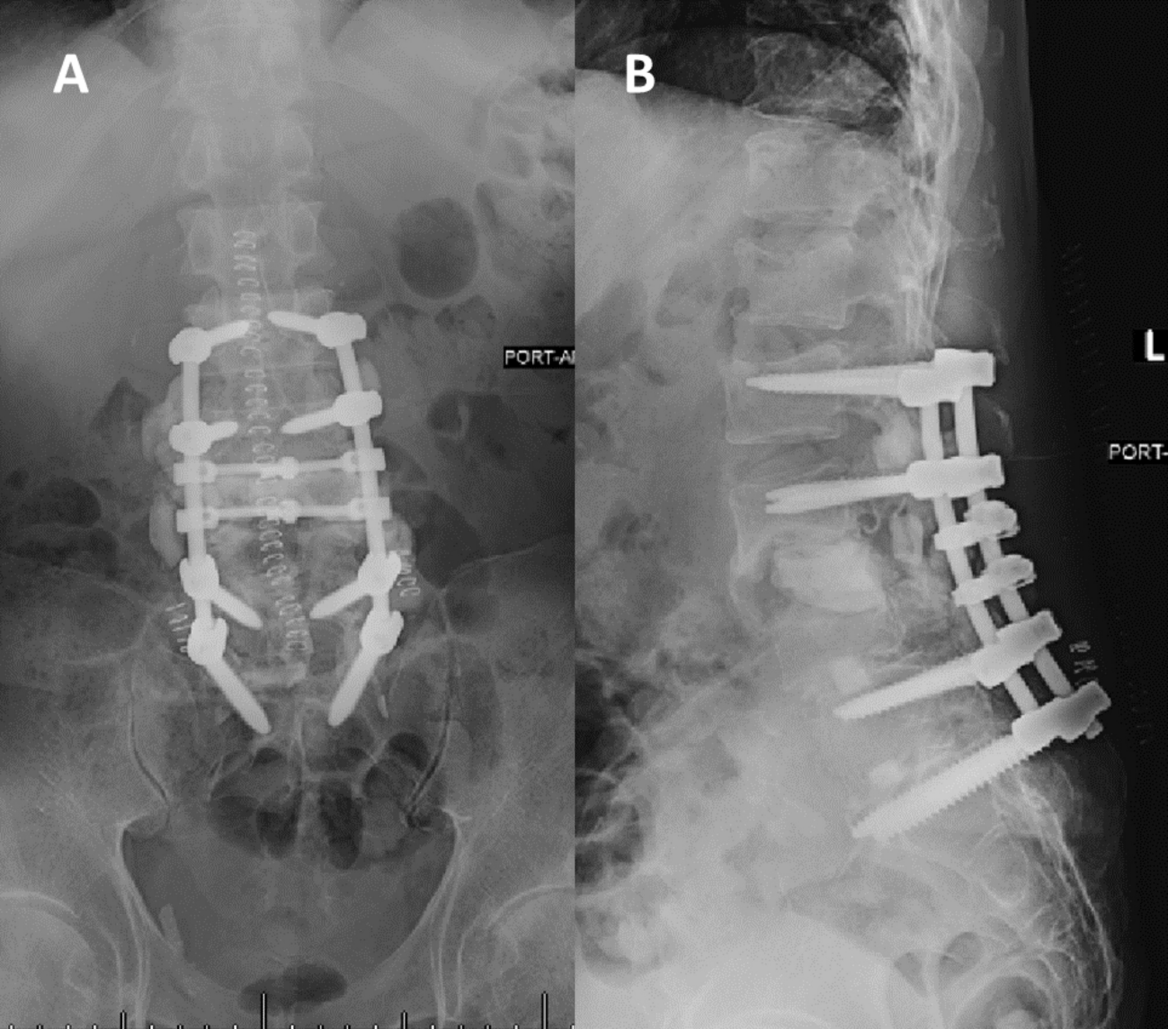
Postoperative lumbar AP (A) and lateral (B) X-rays Removing of the existing hardware, including the L3-4 XLIF, was followed by instrumentation from L2-S1 and the placement of a cement spacer in the L3-4 disc space. XLIF: extreme lateral interbody fusion; AP: antero-posterior

In light of the acute infection resulting in segmental instability, the patient was planned for a two-stage intervention. In the first stage, removal of his existing L4-S1 posterior hardware was followed by spinal canal decompression, which allowed the retrieval of the loose L3-4 interbody loose implant as well as multiple tissue samples for culture and pathology. Spinal stabilization was achieved by placing antibiotic-impregnated temporary polymethyl-methacrylate (PMMA) spacer in the L3-4-disc space and posterior spinal instrumentation from L2 to S1 (Figure 3).

In addition, the placement of antibiotic-impregnated PMMA beads allowed for the optimization of local control of the infection, whereas intravenous empirically administered ceftriaxone and vancomycin enabled systemic control. Specimens taken intraoperatively for aerobes and anaerobes cultures and gram stain were negative. Surprisingly, several separate fungus smears have yielded yeast, resulting in adjusting treatment to oral fluconazole only.

**Figure 4:**
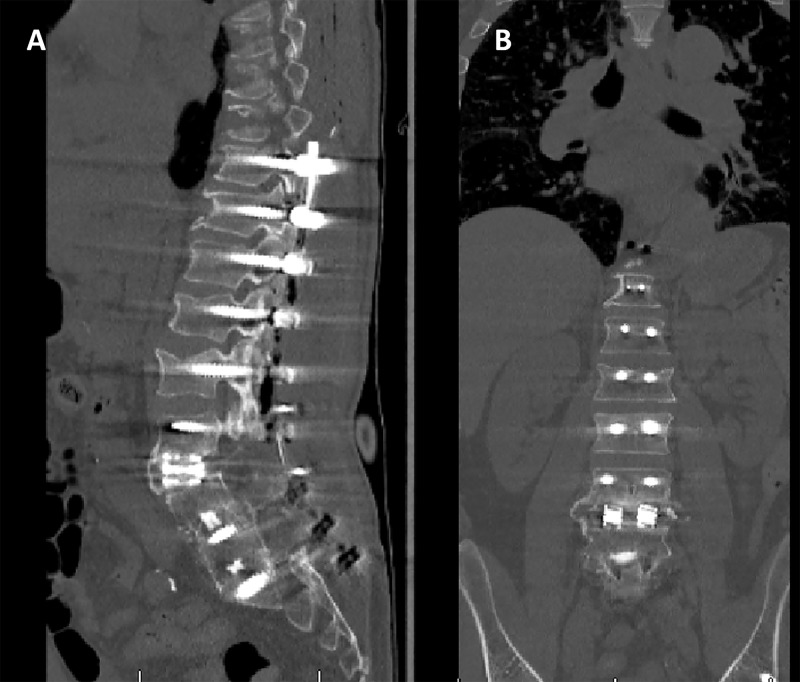
Coronal (A) and sagittal (B) CT images of the lumbar spine following the second stage The temporary PMMA spacer was replaced by an interbody fusion and the previous instrumentation extended to T10 and to the pelvis. PMMA: polymethyl-methacrylate; CT: computed tomography

Following the uneventful surgery, the patient’s back pain and ambulation had progressively improved and the patient was discharged home. The complete resolution of his symptoms as well as persistently negative CRP and ESR at ambulatory follow-up suggested that his infection had resolved. Four months after the first stage, the patient was taken back to the operating room for a planned second stage. The removal of the PMMA spacer and beads and irrigation was followed by a definite fusion of both the L3-4 segment as well as from T10 to his pelvis (Figure 4).

## Discussion

Interbody fusion with a cage performed by the extreme lateral approach (XLIF) has become popular in recent years for the treatment of various degenerative, traumatic, and deformity conditions affecting the spine. Similar to other MIS techniques, the procedure is not without risk, with the most commonly reported complications being neurological deficits and anterior thigh pain [4]. Surgical site infection (SSI) is yet another possible major complication, often demanding a revision surgery and a prolonged hospital stay. While the incidence of SSI in open approaches has been estimated to be between 1.9% and 5.5% [5-6], MIS has been associated with a six-fold rate decrease [2]. A study looking specifically at the infections rate associated with the lateral approach found comparable low infections rates, with 0.27% and 0.14% rates of superficial and deep wound infections, respectively [7].

The most common pathogen to cause deep infection and vertebral osteomyelitis following spinal instrumentation is Staphylococcus aureus followed by Escherichia coli and Enterococcus faecalis [3]. Fungal infections of the spine are uncommon, usually affecting patients who are immunocompromised secondary to diabetes mellitus, chemotherapy, chronic corticosteroid use, or malnutrition. Fungal vertebral osteomyelitis following spinal surgery is an extremely rare occurrence, requiring a high clinical index of suspicion as one-third of patients with candida-related spondylitis lack fever and lab tests are usually non-specific [8].

## Conclusions

In this case, a history of chronic alcohol abuse with relative malnutrition probably played a role in the patient’s pathogenesis, leading to the extremely rare occurrence of fungal osteomyelitis following an MIS lateral approach intervertebral fusion. In conclusion, we suggest that in the presence of the above-mentioned patient-related risk factors, a fungal infection should be considered in the differential diagnosis, regardless of the approach and extent used.
